# Associations of spleen volume with markers of blood count and lipid profile in a large population-based study

**DOI:** 10.48101/ujms.v128.9785

**Published:** 2023-09-15

**Authors:** Muhammad Naeem, Till Ittermann, Marcello Ricardo Paulista Markus, Mohammed Farah Mahmoud Mousa, Laura von Heder, Robin Bülow, Marcus Dörr, Matthias Nauck, Ali Agdassi, Florian H. Heidel, Henry Völzke

**Affiliations:** a Institute for Community Medicine, University Medicine Greifswald, Germany; b Department of Internal Medicine B – Cardiology, Intensive Care, Pulmonary Medicine and Infectious Diseases, University Medicine Greifswald, Germany; c Institute for Radiology and Neuradiology, University Medicine Greifswald, Germany; d Institute for Clinical Chemistry and Laboratory Medicine, University Medicine Greifswald, Germany; e Department of Internal Medicine A, University Medicine Greifswald, Germany; f Department of Internal Medicine C, University Medicine Greifswald, Germany; g Hematology, Hemostasis, Oncology and Stem Cell Transplantation, Hannover Medical School (MHH), Germany; h Department of Zoology, University of Malakand, 18800, Pakistan

**Keywords:** spleen volume, MRI, blood count, lipid profile

## Abstract

**Background:**

The aim of our study was to investigate associations of spleen volume with blood count markers and lipid profile in the general population.

**Materials & methods:**

Cross-sectional data from 1,106 individuals aged 30–90 years from the population-based Study of Health in Pomerania (SHIP-START-2) were analyzed. Blood count markers included red blood cell (RBC) counts, hemoglobin, platelet count, and white blood cell (WBC) counts. Lipid profile included total-cholesterol, high-density lipoprotein-cholesterol (HDL-C), and low-density lipoprotein-cholesterol (LDL-C) as well as triglycerides. Linear regression models adjusted for age, sex, body height, and weight were used to associate standardized spleen volume with blood counts and lipid profile markers.

**Results:**

Spleen volume was positively associated with RBC (*β* = 0.05; 95% confidence interval [CI] = 0.03 to 0.08) and hemoglobin (β = 0.05; 95% CI = 0.01 to 0.09) but inversely with platelet count (β = −16.3; 95% CI = –20.5 to −12.1) and WBC (β = −0.25; 95% CI = −0.37 to −0.14). Furthermore, spleen volume showed inverse associations with total cholesterol (β = −0.17; 95% CI = −0.24 to −0.09), HDL-C (β = −0.08; 95% CI = −0.10 to −0.05), and LDL-C (β = −0.12; 95% CI = −0.17 to −0.06). There was no significant association of spleen volume with triglycerides.

**Conclusion:**

Our study showed that the spleen volume is associated with markers of the blood count and lipid profile in the general population.

## Introduction

The spleen is the second largest lymphoid organ in the body and consists of the two parts, white and red pulp (1). While the white portion produces immunological response against infection and inflammation, the red pulp stores and removes old blood cells and serves as filter against bacteria or microorganism (2). Men have comparatively a larger mean spleen volume than women, and it was found that spleen volume is correlated with body height and weight (3, 4).

There are several studies from selected populations or animal models describing associations between spleen size and blood parameters. Spleen function regulates the abundance of circulating platelets, and it has been shown that splenectomized individuals are prone to thromboembolism (5). One study reported that a larger spleen volume measured by computed tomography was related with a lower platelet count, while a positive association between spleen volume and hemoglobin levels was found (6). Similarly, a previous longitudinal study showed that increased splenic metabolic activity after an acute coronary syndrome is associated with pro-inflammatory remodeling of circulating leukocytes and arterial inflammation and is an independent predictor of subsequent cardiovascular events (7). A pilot study conducted in a small group of selected patients showed that spleen volume was inversely associated with the white blood cell (WBC) count (8). In line with this, experimental models showed that levels of red blood cell (RBC) count and hemoglobin were higher in splenectomized mice compared to non-splenectomized controls (9).

There is limited knowledge regarding the association of spleen volume with lipid markers. An experimental study conducted in splenectomized rats suggests that the spleen may alter circulating lipid levels (10). While an experimental study in mice showed that total cholesterol and low-density lipoprotein (LDL)-cholesterol levels were higher, and high-density lipoprotein (HDL)-cholesterol levels were lower in splenectomized mice compared to a control group (11). Another study showed that auto transplantation of the spleen may normalize lipid levels (12). Another experimental study in apolipoprotein E-deficient mice reported no significant effect of splenectomy on lipid metabolism after treatment with an atherogenic diet (13).

Previous literature on the effects of the spleen on blood count and lipid levels mostly focused on splenectomized patients or animal models (9, 10, 13, 14). There is a lack of studies that evaluated associations of spleen volume with parameters of blood count or lipid metabolism in the general population. Thus, in the present study, we aim to investigate associations of spleen volume derived from magnetic resonance imaging (MRI) with markers of the blood count and lipid metabolism in a population-based sample.

## Methods

### Study design and population

This study is based on data from the second follow-up of the population-based Study of Health in Pomerania (SHIP-START) conducted in Northeast Germany. At baseline of SHIP-START, 6,267 eligible subjects were randomly selected from population registries. Of those, 4,308 individuals were examined between 1997 and 2001 (response 68.8%). In the present analyses, we used data from the second follow-up of SHIP-START-2, in which 2,333 individuals aged 30–93 years were examined between 2008 and 2012. All participants gave an informed written consent, and both studies followed the recommendations of the Declaration of Helsinki and were approved by the Ethics Committee of the University of Greifswald.

Of the 2,333 individuals participating in SHIP-START-2, 1,306 underwent the MRI examination. After exclusion of 197 individuals with missing data in the spleen sequence and three individuals with a splenectomy, the study population consisted of 1,106 individuals.

### Assessments

Fasting blood samples were collected from the cubital vein of participants in the supine position between 7 am and 1 pm All samples were either analyzed immediately or stored at − 80°C. Serum lipids (total cholesterol, HDL-cholesterol, LDL-cholesterol, and total triglycerides) were measured in serum using the Dimension Vista 500 analytical system (Siemens AG, Eschborn, Germany). Glucose levels were measured using a hexokinase method (Dimension Vista, Siemens Healthcare Diagnostics, Eschborn, Germany). Measurements of the blood count including RBC, hemoglobin, mean corpuscular volume (MCV), mean corpuscular hemoglobin (MCH), platelet count, and WBC were determined at maximum two hours after obtaining sample in anticoagulated whole blood on the Sysmex platform XE 5000 (Sysmex Corporation, Kobe, Japan).

All participants were asked to bring all of their medications to the examination center. Medication data were obtained online using the IDOM software (online drug-database leaded medication assessment) and categorized according to the Anatomical Therapeutical Chemical (ATC) classification index. Lipid-lowering medication was defined by the ATC code C10.

### Spleen volume assessment

Axial acquired diffusion-weighted MRI of the upper abdomen was performed on a 1.5-T MRI system (Magnetom Avanto; Siemens Medical Systems, Erlangen, Germany) using a 12-channel phased-array surface coil with subjects in a supine position. The isotropic diffusion-weighted imaging was performed using a spin-echo-based echo-planar imaging sequence. Imaging series with different diffusion weightings (b-values) were acquired using b-values of 50 mm^2^/s, 400 mm^2^/s, and 800 mm^2^/s. The acquisition was gated using a prospective acquisition correction technique and following imaging parameters: repetition/echo time = 4,140/72 [ms], field of view = 284 × 379 [mm^2^], a matrix of 192 × 115, a voxel size of 2.0 × 2.0 × 6.0 [mm], a slice gap of 1.2 [mm], a flip angle of 90°, and a bandwidth of 1,735 Hz/Pixel. Quantitative image analysis of all b-800 images was performed by one observer after training, and interobserver certification was computed together with a radiology resident with 5 years of abdominal MRI experience. Mean interobserver variability was 1.30% (mean ± 1.96 standard deviation: −7.75 to 10.35; interclass correlation coefficient: 0.99) in a random subsample of 20 images. The volume calculation was performed by summation of each contoured spleen slice areas with the slice thickness plus slice gap by MeVisLab®-Software (MeVis Medical Solutions AG, Bremen, Germany) after conversion of the acquired DICOM data to the NIfTI standard (Neuroimaging Informatics Technology Initiative) (15).

### Statistical analysis

Stratified by spleen volume, data were expressed as median (25th and 75th percentiles) for continuous data and as absolute numbers (percentages) for categorical data. Associations of spleen volume with markers of blood count and lipid profile were calculated by regression models. Linear regression was applied to continuous outcomes and logistic regression to dichotomous outcomes. All models were adjusted for age, sex, body height, and body weight. To account for the drop-out from the main examinations in SHIP-START-2 to the MRI examinations, we calculated inverse probability weights by logistic regression with participation at the MRI examinations as outcome and health-related markers from the main examinations as explanatory variables. These weights were used in all regression analyses. A *P* < 0.05 was considered as statistically significant. All analyses were conducted with Stata 16.1 (Stata Corporation, College Station, TX, USA).

## Results

Table 1 shows the general characteristics of the study population stratified into the groups lower (below median) and larger spleen volume (above median). The study population consisted of 1,106 individuals, of which 529 were men (48%) and 577 were women (52%). Subjects in the lower spleen volume group were in median older and had higher levels of MCV, MCH, platelet count, total cholesterol, and HDL-cholesterol with a more frequent use of lipid lowering medications than individuals in the larger spleen volume group. Furthermore, individuals in the larger spleen volume group had higher median values of BMI, RBC, and hemoglobin and were more likely to be former smoker compared to individuals in the lower spleen volume group.

**Table 1:** Characteristics of study populations stratified by percentiles of spleen volume.

Variables	Spleen volume			
	Below 50th percentile (*n* = 554)		Above 50 percentile (*n* = 552)	
	Men (*n* = 265)	Women (*n* = 289)	Men (*n* = 264)	Women (*n* = 288)
Age, years	57.0 (46.0; 67.0)	57 (47; 65)	54 (43; 65)	54 (43.5; 64.5)
Body mass index, kg/m^2^	26.8 (25.1; 29.8)	25.0 (22.8; 28.5)	28.6 (26.3; 31.4)	27.8 (24.7; 31.8)
Alcohol consumption, g/day	10.1 (4.13; 20.2)	3.30 (0.67; 7.60)	9.00 (2.46; 22.8)	2.61 (1.31; 5.68)
Smoking status (%)				
Never smoker	23.8	51.2	31.8	46.5
Former smoker	50.8	27.0	54.2	37.2
Current smoker	25.7	21.8	14.0	16.3
Red blood cell count, 10^12^/L	4.70 (4.50; 5.00)	4.30 (4.20; 4.60)	4.90 (4.60; 5.10)	4.45 (4.20; 4.70)
Hemoglobin, mmol/L	9.00 (8.60; 9.50)	8.20 (7.80; 8.60)	9.10 (8.70; 9.60)	8.30 (7.90; 8.60)
Mean corpuscular volume, fl	92.1 (89.4; 94.4)	91.5 (89.4; 93.9)	88.9 (86.9; 92.1)	90.5(87.9; 92.8)
Mean corpuscular hemoglobin, fmol	1.91 (1.85; 1.97)	1.89 (1.82; 1.94)	1.88 (1.83; 1.94)	1.87 (1.81; 1.93)
Platelet count, 10^9^/L	216 (183; 254)	244 (214; 287)	197 (166; 226)	229 (195; 259)
White blood cell count, 10^9^/L	5.72 (4.81; 6.80)	5.83 (4.89; 6.94)	5.44 (4.69; 6.55)	5.83 (4.87; 6.97)
Total cholesterol, mmol/L	5.60 (4.80; 6.20)	5.70 (4.90; 6.50)	5.20 (4.60; 5.90)	5.30 (4.60; 6.00)
LDL-cholesterol, mmol/L	3.55 (2.82; 4.00)	3.40 (2.78; 4.20)	3.23 (2.73; 3.80)	3.16 (2.60; 3.77)
HDL-cholesterol, mmol/L	1.37 (1.14; 1.58)	1.61 (1.40; 1.92)	1.20 (1.00; 1.41)	1.45 (1.27; 1.72)
Triglycerides, mmol/L	1.68 (1.18; 2.49)	1.27 (0.92; 1.85)	1.84 (1.29; 2.71)	1.38 (0.96; 2.12)
Lipid-lowering medication (%)	14.2	18.5	13.9	16.7

Data are expressed as median (25th and 75th percentiles) for continuous data and as absolute numbers (percentages) for categorical data.

In multivariable linear regression models adjusted for age, sex, body height, and weight, we found significant associations of spleen volume with blood count markers (Table 2; Figure 1a to d). We observed positive associations of the spleen volume with RBC and hemoglobin in the whole population as well as in males and females. Moreover, we found inverse associations of spleen volume with MCV, platelet count, and WBC in the whole population as well as in males and females. Spleen volume was inversely associated with MCH in the whole population and in males but not in females.

**Table 2:** Associations between spleen volume and blood count markers.

Variables	Spleen volume (mL)		
	All	Men	Women
	β (95% CI)	β (95% CI)	β (95% CI)
Red blood cell count, 10^12^/L	0.05 (0.03; 0.08)*	0.06 (0.03; 0.99)*	0.03 (−0.01; 0.07)
Hemoglobin, mmol/L	0.05 (0.01; 0.09)*	0.06 (0.00; 0.12)*	0.03 (−0.31; 0.10)
Mean corpuscular volume, fl	−1.01 (−1.37; −0.66)*	−1.12 (−1.65; −0.60)*	−0.81 (−1.27; −0.36)*
Mean corpuscular hemoglobin, fmol	−0.01(−0.02; −0.00)*	−0.01 (−0.02; 0.00)*	−0.01 (−0.02; 0.00)
Platelet count, 10^9^/L	−16.3 (−20.5; −12.1)*	−13.5 (−19.1; −7.83)*	–20.8 (–25.7; –15.8)*
White blood cell count, 10^9^/L	−0.25 (−0.37; −0.14)*	−0.24 (−0.40; 0.08)*	−0.26 (−0.42; −0.10)*

β coefficients were derived from linear regression models adjusted for age, sex, body height, and weight. Analyses are weighted for drop-out to the MRI examinations; β coefficients are reported by one standard deviation (80 mL) for continuous spleen volume as exposure.

* *P* < 0.05.

**Figure 1a:**
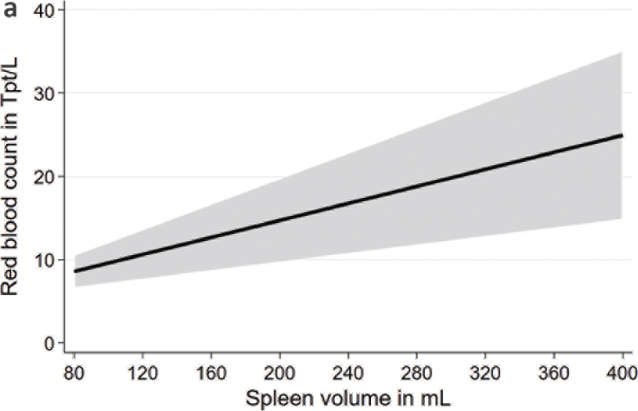
Associations between spleen volume and red blood cell count based on linear regression after adjustment for age, sex, body height, and weight.

**Figure 1b:**
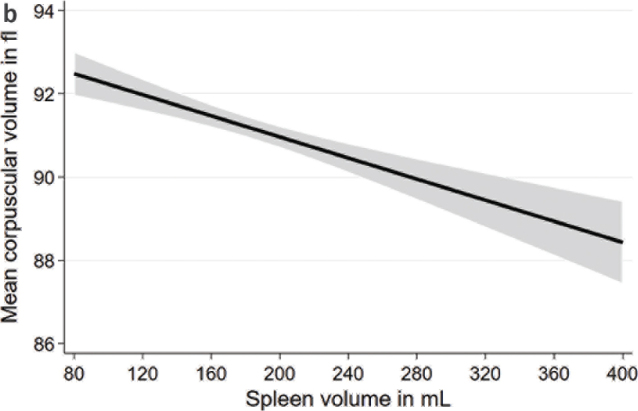
Associations between spleen volume and mean corpuscular volume based on linear regression after adjustment for age, sex, body height, and weight.

**Figure 1c:**
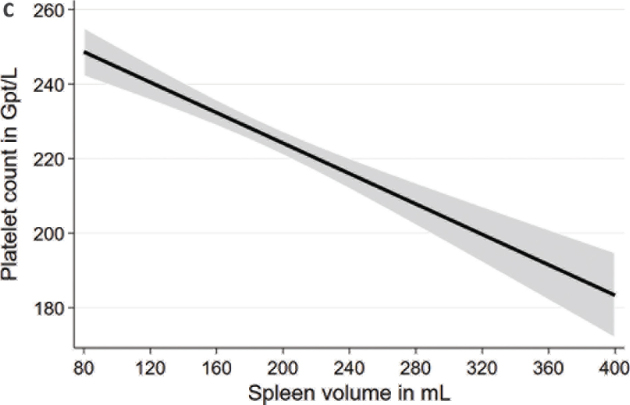
Associations between spleen volume and platelet count based on linear regression after adjustment for age, sex, body height, and weight.

**Figure 1d:**
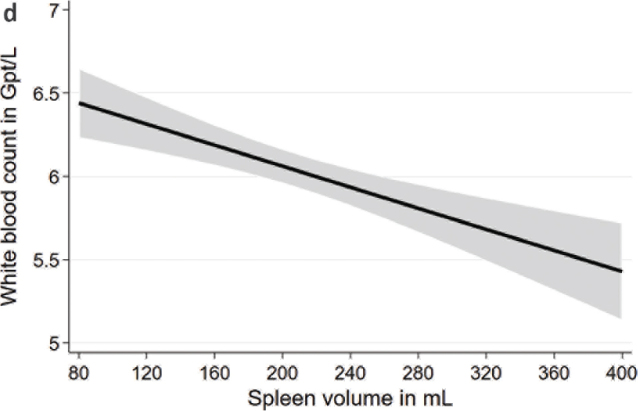
Associations between spleen volume and white blood cell count based on linear regression after adjustment for age, sex, body height, and weight.

After adjustment for age, sex, body height, and weight, spleen volume was inversely associated with total cholesterol and LDL-cholesterol in the whole population as well as in males and females (Table 3; Figure 2a). Spleen volume was inversely associated with HDL-cholesterol in the total population and in males but not in females (Figure 2b). Spleen volume was not significantly associated with TG levels, intake of lipid lowering medication, and dyslipidemia.

**Table 3:** Associations between spleen volume and lipids markers.

Variables	Spleen volume (mL)		
	All	Men	Women
	β (95% CI)	β (95% CI)	β (95% CI)
Total cholesterol, mmol/L	−0.17 (−0.24; −0.09)*	−0.13 (−0.22; −0.04)*	−0.24 (−0.36; −0.13)*
LDL-cholesterol, mmol/L	−0.12 (−0.17; −0.06)*	−0.09 (−0.16; −0.03)*	−0.15 (−0.25; −0.05)*
HDL-cholesterol, mmol/L	−0.08 (−0.10; −0.05)*	−0.06 (−0.09; −0.04)*	−0.10 (−0.14; −0.06)
Triglycerides, mmol/L	0.15 (−0.01; 0.30)	0.16 (−0.06; 0.38)	0.12 (−0.05; 0.28)
	**Odds ratio (95% CI)**	**Odds ratio (95% CI)**	**Odds ratio (95% CI)**
Intake of lipid lowering medication	0.91 (0.73; 1.13)	0.92 (0.70; 1.20)	0.89 (0.63; 1.27)
Dyslipidemia	0.92 (0.79; 1.08)	0.93 (0.77; 1.13)	0.88 (0.67; 1.34)

LDL: low-density lipoprotein; HDL: high-density lipoprotein.

β coefficients were derived from linear regression; odds ratio were derived from logistic regression models. Models were adjusted for age, sex, body height, and weight. Analyses are weighted for drop-out to the MRI examinations; β coefficients are reported by one standard deviation (80 mL) for continuous spleen volume as exposure.

* *P* < 0.05.

**Figure 2a:**
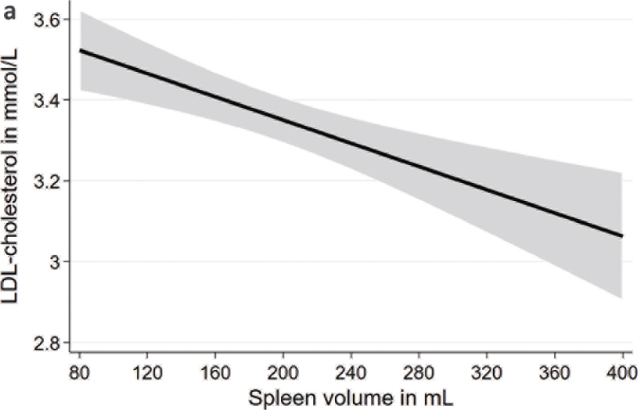
Associations between spleen volume and LDL-cholesterol based on linear regression after adjustment for age, sex, body height, and weight. LDL, low-density lipoprotein.

**Figure 2b:**
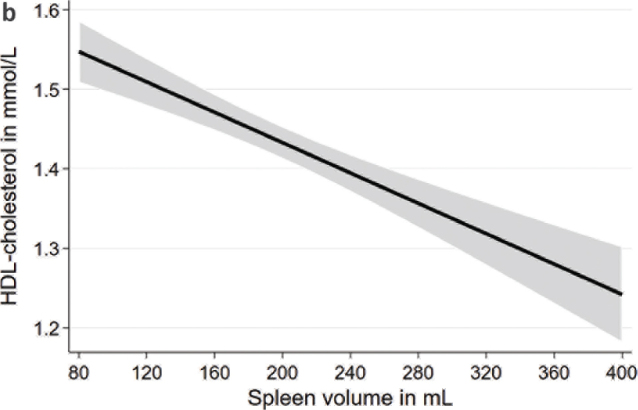
Associations between spleen volume and HDL-cholesterol based on linear regression after adjustment for age, sex, body height, and weight. HDL, high-density lipoprotein.

In sensitivity analyses, we excluded individuals using lipid lowering medication, which did not change our results.

## Discussion

To the best of our knowledge, there is no study that investigated associations of MRI-derived spleen volume with markers of blood count and lipid metabolism in the general population. We demonstrated positive associations of spleen volume with RBC and hemoglobin, while spleen volume was inversely associated with MCH, MCV, platelet count, and WBC. These associations were consistent in the whole population as well as in males and females except for MCH, where we observed no significant association in females. Regarding lipids, we showed that spleen volume was inversely associated with total cholesterol, LDL-cholesterol, and HDL-cholesterol. These associations were independent of age, sex, body height, and weight.

Similar to our findings, a previous study conducted in small group of healthy individuals also found positive associations of spleen volume with hemoglobin and RBC as well as inverse associations of spleen volume with platelet count and lymphocytes percentage (6). A comparable result was also obtained from a small group of patients where an inverse association of spleen volume with WBC was observed (8). Our finding is also supported by previous findings from experimental models, in which non-splenectomized mice had higher levels of RBC and hemoglobin compared to splenectomized mice (9). However, compared to the aforementioned studies, which were conducted on small groups of patients and healthy individuals or using experimental animal models, we used a large sample size of individuals selected from a general population. Although one of the previous studies used CT for spleen volume assessment (6), we used MRI for spleen volume measurement, which is a more accurate and sensitive marker than CT or ultrasonography (16).

The underlying mechanisms for the association of spleen volume with the blood counts are largely unclear. In hematopoietic cancers, larger spleen volume can be associated with extramedullary hematopoiesis and systemic inflammation. However, in healthy individuals, extramedullary hematopoiesis is neither present to a relevant extent nor responsible for higher spleen size. Recent reports, however, have indicated the presence of clonal somatic aberrations that increase with age (17), mediate systemic inflammation (18), and may contribute to both larger spleen size and RBC aberrant blood cell numbers (19, 20). We also speculate that the role of exchangeable platelets spleen pools might explain our results. Compared to other blood cells, about one-third of total mass of platelets are sequestered in spleen (5). With increase in spleen size, the sequestration of platelets increases, which might reduce the number of circulating platelets (21). The loss of spleen volume following splenectomy can increase the numbers of platelets and their activity and may cause portal thrombosis (14).

Our results showed that a lower spleen volume was associated with higher lipid levels. Similar to our findings, previous results from rat models also showed a change in lipid levels after splenectomy (10–12). For instance, one experimental study in rats showed that auto-transplantation of the spleen normalized lipid levels that were previously elevated (10). Moreover, levels of total cholesterol and LDL-cholesterol increased and HDL-cholesterol decreased in rats after splenectomy (12). For total and LDL-cholesterol, our results agree with those from the animal models, but we observed higher HDL-cholesterol levels in individuals with a lower spleen volume, which is in contrast to the findings from the animal models. The difference in results might be related to differences between humans and animals. Furthermore, a splenectomy cannot be directly compared with differences in spleen volume. Further studies in the general population as well as in selected patient populations are warranted to verify and extend the findings of our study.

The spleen is considered a reservoir for lipid and lipoprotein lipase enzymes and is responsible for the storage and transportation of lipids (22). An increase in spleen size may stimulate lipoprotein lipase activity (LPL), thus enhancing cholesterol disposition in the spleen resulting in hypolipidemia. Another possibility is that larger spleen may contain greater amount of LPL enzymes without changing their activity (23). However, we can speculate that a change in spleen volume can affect the levels of lipids in the spleen and, hence, the overall blood plasma pool. Another proposed mechanism might be a splenic factor involved for lipid metabolism in the spleen (24). During splenomegaly, the splenic factor induces the phagocytosis activity that enhances the accumulation of lipid molecules in the spleen, thus decreasing serum lipid levels (12).

The large number of individuals recruited in a population-based setting is one of the strengths of our study. We used MRI as a standardized method for the estimation of spleen volume. The limitation of our study is the cross-sectional design, which did not allow us to draw casual inference. Likewise, as indicated earlier, the SHIP-cohort investigated in this analysis has not been investigated for the presence of clonal hematopoiesis, which is a frequent finding in individuals above the age of 60 (17). Clonal hematopoiesis can contribute to a systemic inflammatory state that contributes to cardiovascular and chronic inflammatory disease (18, 25). Therefore, we cannot exclude the influence of clonal hematopoiesis on blood counts and spleen size in this study population.

## Conclusions

Our study showed that the spleen volume is associated with markers of the blood count and lipid profile. A larger spleen volume was associated with higher levels of RBC and hemoglobin, but with a lower platelet count, a lower WBC, and lower lipid levels. Further studies are warranted to validate and explain our findings.
